# Transfer of the cytochrome P450-dependent dhurrin pathway from *Sorghum bicolor* into *Nicotiana tabacum* chloroplasts for light-driven synthesis

**DOI:** 10.1093/jxb/erw067

**Published:** 2016-03-11

**Authors:** Thiyagarajan Gnanasekaran, Daniel Karcher, Agnieszka Zygadlo Nielsen, Helle Juel Martens, Stephanie Ruf, Xenia Kroop, Carl Erik Olsen, Mohammed Saddik Motawie, Mathias Pribil, Birger Lindberg Møller, Ralph Bock, Poul Erik Jensen

**Affiliations:** ^1^Copenhagen Plant Science Centre, Center for Synthetic Biology bioSYNergy, Villum Research Center “Plant Plasticity”, Department of Plant and Environmental Sciences, University of Copenhagen, Thorvaldsensvej 40, DK-1871 Frederiksberg C, Copenhagen, Denmark; ^2^Max Planck Institute of Molecular Plant Physiology, Potsdam-Golm, Germany

**Keywords:** Cytochrome P450, chloroplast, dhurrin pathway, light-driven, photosynthesis, thylakoids.

## Abstract

Plant chloroplasts are light-driven cell factories that have great potential to act as a chassis for metabolic engineering applications. Using plant chloroplasts, we demonstrate how photosynthetic reducing power can drive a metabolic pathway to synthesise a bio-active natural product. For this purpose, we stably engineered the dhurrin pathway from *Sorghum bicolor* into the chloroplasts of *Nicotiana tabacum* (tobacco). Dhurrin is a cyanogenic glucoside and its synthesis from the amino acid tyrosine is catalysed by two membrane-bound cytochrome P450 enzymes (CYP79A1 and CYP71E1) and a soluble glucosyltransferase (UGT85B1), and is dependent on electron transfer from a P450 oxidoreductase. The entire pathway was introduced into the chloroplast by integrating *CYP79A1*, *CYP71E1*, and *UGT85B1* into a neutral site of the *N. tabacum* chloroplast genome. The two P450s and the UGT85B1 were functional when expressed in the chloroplasts and converted endogenous tyrosine into dhurrin using electrons derived directly from the photosynthetic electron transport chain, without the need for the presence of an NADPH-dependent P450 oxidoreductase. The dhurrin produced in the engineered plants amounted to 0.1–0.2% of leaf dry weight compared to 6% in sorghum. The results obtained pave the way for plant P450s involved in the synthesis of economically important compounds to be engineered into the thylakoid membrane of chloroplasts, and demonstrate that their full catalytic cycle can be driven directly by photosynthesis-derived electrons.

## Highlight

A pathway containing two cytochrome P450s and a glucosyltransferase has been stably expressed in *Nicotiana tabacum* chloroplasts. The functional P450s are enriched in the thylakoids and receive electrons from photo-reduced ferredoxin.

## Introduction

Chloroplasts are light-driven cell factories that provide energy, reducing power, and carbon building blocks for a plethora of pathways (Bock and Warzecha, 2010). The chloroplast genome of *Nicotiana tabacum* was one of the first organelle genomes to be sequenced (Shinozaki, 1986). The presence of multiple copies of the chloroplast (plastid) genome within a single chloroplast, straightforward stable genetic transformation methods that rely on homologous recombination, the absence of epigenetic silencing, and position effects makes the chloroplast a highly suitable chassis for synthetic biology applications (Sanford, 1990; Bock, 2001, 2014, 2015; Maliga, 2004). In addition, the availability of unique regulatory tools like synthetic riboswitches (Verhounig *et al.*, 2010; Emadpour *et al.*, 2015) and intercistronic expression elements (IEEs) facilitate inducible transgene expression and efficient multigene engineering from synthetic operons in the chloroplast (Lu *et al.*, 2013; Zhou *et al.*, 2007).

Currently, plant chloroplasts are being developed as chassis for the production of vaccines, antibiotics, wax esters, vitamins, and plant-specialized metabolites (Zhou *et al.*, 2008; Bock and Warzecha, 2010; Lu *et al.*, 2013; Aslan *et al.*, 2014; Lassen *et al.*, 2014; Gnanasekaran *et al.*, 2015;). In this context, the biosynthesis of specialized metabolites in plants often involves cytochrome P450 enzymes (P450s). P450s are heme-containing enzymes that catalyse an array of reaction types, including C- and N-hydroxylation, O-, N-, and S-dealkylation, sulfoxidation, desaturation, deformylation, epoxidation, deamination, desulfurization, dehalogenation, peroxidation, and N-oxide reduction (Schlichting, 2000; Hannemann *et al.*, 2007). Eukaryotic P450s are membrane bound, typically to the endoplasmic reticulum (ER) or inner mitochondrial membranes, through a single N-terminal transmembrane-spanning segment (Werck-Reichhart and Feyereisen, 2000). Recently, engineering of P450-dependent pathways into chloroplasts has gained increased attention due to the possibility of driving the catalytic activity of P450s by directly using photosynthetic reducing power via photo-reduced ferredoxin (Fd). This approach eliminates the need for P450 oxidoreductase (POR) and NADPH as the electron donor for the P450s (Jensen *et al.*, 2011a; Nielsen *et al.*, 2013; Lassen *et al.*, 2014).

In this study, we stably integrated three genes encoding enzymes for the biosynthetic pathway of dhurrin into the chloroplast genome of tobacco with the purpose of exploiting the photosynthetic reducing power to drive the P450s, using the tyrosine and activated sugar substrates provided endogenously by chloroplast metabolism. Dhurrin is a cyanogenic glucoside in *Sorghum bicolor* (Tattersall *et al.*, 2001). The biosynthetic pathway involves two cytochromes P450s (CYP79A1 and CYP71E1) and a POR bound to the endoplasmic membrane system via their respective transmembrane domain and a soluble glucosyltransferase UGT85B1 (Møller and Seigler, 1999). CYP79A1 catalyses conversion of the substrate tyrosine into (*Z*)-*p*-hydroxyphenylacetaldoxime (Sibbesen *et al.*, 1995), whereas CYP71E1 mediates conversion of *p*-hydroxyphenylacetaldoxime (oxime) into the cyanohydrin *p*-hydroxymandelonitrile with *p*-hydroxyphenylacetonitrile as an intermediate (nitrile) (Kahn *et al.*, 1997). The catalytic activity of P450s is dependent on electron transfer from NADPH mediated by the POR (Jensen and Moller, 2010). In the final step of the pathway, *p*-hydroxymandelonitrile is converted into the cyanogenic glucoside dhurrin by the glucosyltransferase UGT85B1 (Jones *et al.*, 1999). The catalytic domains of the membrane-bound enzymes face the cytosol and are, together with the UGT85B1, localized in a metabolon (Nielsen *et al.*, 2008; Møller, 2010). Each single catalytic step within the dhurrin pathway is well characterized and can be easily monitored, and the pathway therefore constitutes an excellent model system for testing metabolic engineering approaches involving P450s.

Metabolic engineering of the entire dhurrin pathway and the individual enzymes from *S. bicolor* has previously been carried out using stable nuclear transformation of *Arabidopsis thaliana* and *N. tabacum* to examine changes in the transcriptome and metabolome patterns in the engineered plants (Bak *et al.*, 2000; Kristensen *et al.*, 2005), and to confer resistance to the flea beetle *Phyllotreta nemorum* (Tattersall *et al.*, 2001). In addition, as a proof-of-concept study, *Agrobacterium tumefaciens*-mediated transient expression of the dhurrin pathway was targeted to chloroplasts of *N. benthamiana* to demonstrate that the reducing power of photosynthesis can be utilized to drive the P450s (Nielsen *et al.*, 2013).

Transient expression of pathways/enzymes in plants by *A. tumefaciens*-mediated transformation is a simple way to study the expression of candidate gene(s) (Bach *et al.*, 2014). However, this technology is limited because it is not possible to maintain direct control of the protein expression levels, as well as by the rapid reduction in the levels of expressed protein 5–7 days after infiltration (Sparkes *et al.*, 2006). In this work, we stably transformed the entire dhurrin pathway into the chloroplast genome of tobacco to investigate whether the chloroplast is a suitably robust chassis for light-driven metabolite synthesis. The genes encoding the entire dhurrin pathway were stably integrated into a neutral site of the chloroplast genome of *N. tabacum*. The results obtained demonstrate that the chloroplast can be used as a robust and efficient chassis for heterologous expression of P450-dependent pathways, resulting in the formation of high-value natural products that is driven directly by the reducing power of photosynthesis.

## Materials and methods

### Plant material and growth conditions

Tobacco (*N. tabacum*) plants were grown under greenhouse conditions (16h light/8h dark cycle; 25°C/20°C; light intensity of 200–600 µmol m^−2^ s^−1^).

### Chloroplast transformation of *N. tabacum*

**Fig. 1.**
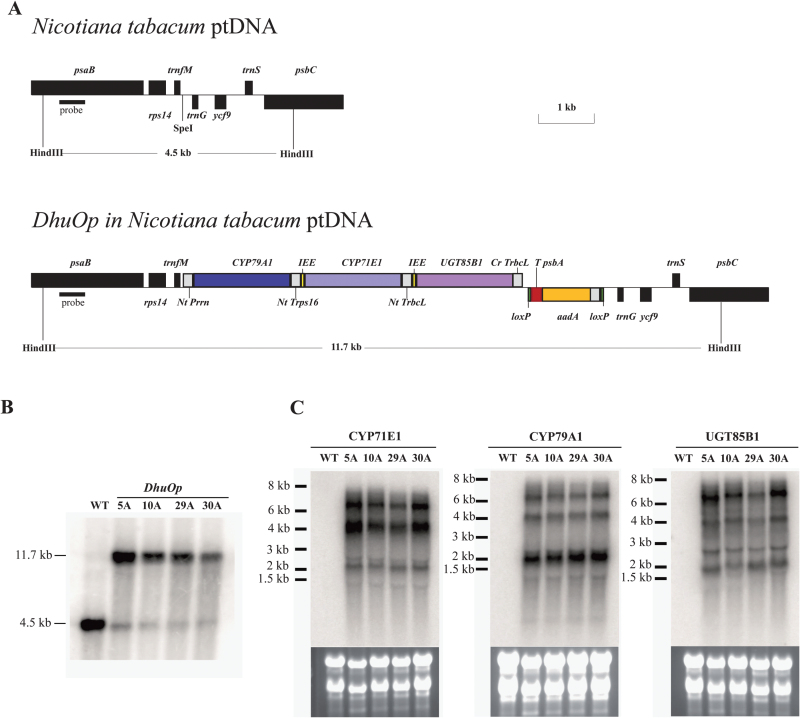
Operon construct (*DhuOp*) used for integration in the chloroplast genome of *N. tabacum*. (**A**) Design of the dhurrin operon constructs (*DhuOp).* The operon is inserted into a unique SpeI site in the intergenic spacer between the *trnfM* and *trnG* genes. The two HindIII restriction sites used for RFLP analysis and the corresponding fragment sizes are also indicated. (**B**) Southern blot analysis performed on the transplastomic lines showing the expected signal at 11.7kb in *DhuOp* lines 5A, 10A, 29A, and 30A. DNA extracted from wild-type *N. tabacum* (WT) was used as a control (4.5kb band). (**C**) Analysis of mRNA accumulation in *DhuOp* lines. The transgene specific hybridization probes detect the processed transcripts of the genes corresponding to the signals in the 2.5kb range and unprocessed bicistronic and tricistronic precursor RNA transcripts corresponding to signal sizes of 4kb and 6kb, respectively. The RNA marker bands are indicated in kilobases (kb).

The three genes comprising the dhurrin pathway and the respective regulatory elements [untranslated regions (UTRs), ribosome binding sites (RBSs), and IEEs] were designed and chemically synthesized (GenScript, USA) in three separate parts (pDHU1, pDHU2, and pDHU3) (Fig. 1A). pDHU1 comprised the P*rrn* promoter (ribosomal RNA operon promoter from *N. tabacum*), the 5′UTR from the phage gene 10 from *Escherichia coli* phage T7 gene, the *CYP79A1* gene, and the 3′UTR from ribosomal protein S16. pDHU2 comprised the IEE to allow endonucleolytic cleavage of the polycistronic precursor transcript into stable monocistronic mRNA molecules (Zhou *et al.*, 2007; Lu *et al.*, 2013), RBS, the *CYP71E1* gene, and the 3′UTR from the ribulose-bisphosphate carboxylase gene (*rbc*L) from *Chlamydomonas reinhardtii*. DHU3 comprised an IEE, RBS, the *UGT85B1* gene, the 3′UTR of the *rbc*L gene from *C. reinhardtii*, and the terminator sequence. All three parts were combined into a tricistronic expression cassette in the pBluescript II SK(+) plasmid to get *DhuOp* by classical ligation. Finally, the construct *DhuOp* from the pBluescript II SK(+) plasmid was subsequently cloned into the tobacco chloroplast transformation vector pKP9 (Zhou *et al.*, 2008) and transformed into the chloroplast genome of *N. tabacum* using the biolistic method. Positive transformants were confirmed by Southern blot analysis and the homoplasmic state of the transplastomic lines was verified by seed germination on RM medium containing 500mg/L spectinomycin (Supplementary Table S1). All the above mentioned procedures were carried out as previously described (Lu *et al.*, 2013).

### Isolation of nucleic acids and hybridization analyses

Total plant DNA was extracted from leaf tissue using a cetyltrimethylammonium bromide-based method (Doyle and Doyle, 1990). Isolated total DNA was digested overnight with HindIII and separated by gel electrophoresis in a 0.8% agarose gel. Subsequently, DNA fragments were transferred onto Hybond XL membranes (GE Healthcare, Little Chalfont, UK). A 550bp amplicon of the *psaB* coding region was generated using primer pair 7247 (5′ CCCAGAAAGAGGCTGGCCC 3′) and 7244 (5′ CCCAAGGGGCGGGAACTGC 3′). The PCR product was purified from gel slices using the Nucleospin Extract II kit (Machery-Nagel, Düren, Germany) and used as a template to generate the restriction fragment length polymorphism (RFLP) probe using the MegaPrime DNA Labeling System (GE Healthcare) with ^32^P-dCTP. For northern blot analysis, total cellular RNA was isolated using the peqGOLD TriFast reagent (Peqlab GmbH, Erlangen, Germany) according to the manufacturer’s specifications. RNA samples were separated in 1% agarose gels under denaturing conditions and transferred to Hybond XL membranes. The coding sequences of CYP79A1, CYP71E1, and UGT85B1 were isolated by restriction digestion from the transformation vector and purified with the Nucleospin Extract II kit (Machery-Nagel). The isolated DNA was radiolabelled by random priming with ^32^P-dCTP using the MegaPrime DNA Labeling System (GE Healthcare).

### Determination of photosynthetic parameters

All photosynthetic parameters were measured using a dual-pulse amplitude modulated fluorometer (DUAL-PAM-100, Heinz Walz GmbH, Germany) according to Maxwell and Johnson (2000) and Baker (2008). After dark adaption for 30min, we simultaneously assessed the state of PSII via chlorophyll fluorescence and the state of PSI via dual wavelength P700 measurements. For this purpose, the leaves were illuminated for 5min with different actinic light intensities (67, 230, or 545 µmol m^−2^ s^−1^). For every light intensity, a saturating pulse of white light (8000 µmol m^−2^ s^−1^ for 0.8s) was applied. The photosynthetic parameters were determined using the DUAL-PAM software.

### Thylakoid and intact chloroplast preparations

Thylakoid membrane and chloroplast samples were prepared from leaves harvested from 5–6-week-old *N. tabacum* plants that had been dark-adapted overnight. All steps were carried out under green safe light (Nielsen *et al.*, 2013). Total chlorophyll and chlorophyll *a/b* ratios of intact leaves were determined using extraction in 96% ethanol and, for chloroplasts and thylakoid fractions, in 80% acetone (Lichtenthaler, 2007).

### SDS-PAGE and immunoblotting analysis

Isolated thylakoids were characterized by SDS-PAGE and western blotting as described by Nielsen *et al.* (2013). Specifically, the nitrocellulose membranes were probed with primary antibodies against CYP79A1, CYP71E1, and UGT85B1 (all diluted 1:2000) or PsaD (1:20 000) in 1% skimmed milk in phosphate-buffered saline-Tween 20 at 4°C overnight. All incubations, washes, and detection were as described in Nielsen *et al.* (2013).

### *In vitro* activity assays with thylakoids

All *in vitro* activity assays with thylakoids (protein amount corresponding to 90 µg chlorophyll) prepared from the *CYP79A1* and *DhuOp* lines were carried out as previously reported (Nielsen *et al.*, 2013) using L-[U-^14^C] tyrosine as the substrate. For the engineered lines expressing only the CYP71E1 enzyme, the activity assays were carried out using 2.6 µM [U-^14^C] (*Z*)-*p*-hydroxyphenylacetaldoxime as the substrate.

### Whole leaf extract preparations for LC-MS analysis

Leaf material was harvested and collected in Eppendorf tubes containing steel balls, frozen in liquid nitrogen, and crushed using a mixer mill. Following addition of pre-cooled 80% methanol (300 µL) and vortexing for 2min, the tubes were incubated at room temperature for 5min to extract the metabolites. The debris was removed by centrifugation (10 000*g*, 5min), and the cleared supernatant was filtered through centrifugal filters (Millipore 0.45 µm) and diluted with deionized water to a final concentration of 20% methanol. All samples for dhurrin analysis were spiked with amygdalin (20ng/µL final concentration) as an internal standard and subjected to LC-MS analysis (Takos *et al.*, 2010).

### Electron microscopic analysis of chloroplast ultrastructure

A minimum set of three fully expanded leaves from wild-type tobacco and each of the transplastomic lines 5C and 29A were examined by TEM. Excised leaf samples (1×3mm) were immediately fixed for 4h in Karnovsky’s fixative (5% glutaraldehyde, 4% paraformaldehyde, 0.1M sodium cacodylate buffer, pH 7.3, 4°C) including a vacuum treatment, washed in cacodylate buffer, and post-fixed in 1% osmium tetroxide (with 0.1M cacodylate buffer) for 8h at 4°C. After washing in buffer and water, the samples were dehydrated in a graded acetone series, infiltrated with increasing ratios of Spurr resin to acetone, and embedded in Spurr resin within flat moulds. The resin was polymerized in an oven at 60°C for 8h. Ultrathin sections (40nm thick) were cut with a diamond knife using a Reichert-Jung/LKB Supernova ultramicrotome and collected on pioloform-coated copper grids. Sections were contrasted with 1% uranyl acetate and lead citrate (2.7% in 3.5% sodium citrate) and examined in a Philips CM 100 TEM at 80kV.

### Localization of chloroplast lipids by confocal microscopy

The lower epidermis was peeled off from fresh leaf pieces and the exposed mesophyll was incubated with Nile Red (0.5mg/mL) for 10min in darkness. To show the distribution of neutral lipids, the specimens were excited with laser light at 514nm in a Leica SP5 X confocal microscope fitted with a 63× water objective and emissions collected within the 538–600nm wavelength range.

## Results

### Construction of a dhurrin operon and transformation into the chloroplast genome of *N. tabacum*

We transformed the entire dhurrin pathway into the chloroplast genome of tobacco. Our aim was to achieve expression of the pathway enzymes in this organelle by overcoming potential limitations in nuclear gene expression, expression stability, translation and/or chloroplast import. The three essential genes (*CYP79A1*, *CYP71E1*, and *UGT85B1*) were linked in a tricistronic operon construct with the essential regulatory elements (Fig. 1A). We named this operon construct *DhuOp*. Successful transformation of the *DhuOp* operon into the plastid genome and integration of the transgene by homologous recombination was confirmed by Southern blot analysis using total DNA extracted from leaf tissue of the transformed lines (Fig. 1A, B). RFLP signals in the transplastomic lines corresponding to the expected size of 11.7kb were observed, confirming the integration of the *DhuOp* operon into the chloroplast genome. Weak RFLP signals of 4.5kb, corresponding to the size of the wild-type fragment, were also observed in the transplastomic lines (Fig. 1A, B). They are very likely derived from so-called promiscuous DNA, plastid DNA fragments present in the nuclear genome (Hager *et al.*, 1999). Such promiscuous DNA fragments have been seen in many previous transplastomic studies (Li *et al.*, 2010; Elghabi *et al.*, 2011; Krech *et al.*, 2013).

The homoplasmic state of the transplastomic lines was verified by germination of seeds on selective synthetic medium containing 500mg/L spectinomycin. All progeny from the transplastomic lines showed uniform resistance against the antibiotic (Supplementary Fig. S1), ultimately confirming that the transplastomic lines were homoplasmic [and thus confirming that the weak wild-type-like hybridization signals indeed originated from promiscuous DNA; for review, see Maliga (2004) and Bock (2015)]. Transcript analysis by northern blot experiments revealed the expected processing of the polycistronic precursor RNA into monocistronic transcripts, but also detected substantial amounts of unprocessed tricistronic and partially processed bicistronic RNAs (Fig. 1C).

### The enzymes of the dhurrin pathway are successfully expressed in the chloroplasts of *N. tabacum*

**Fig. 2.**
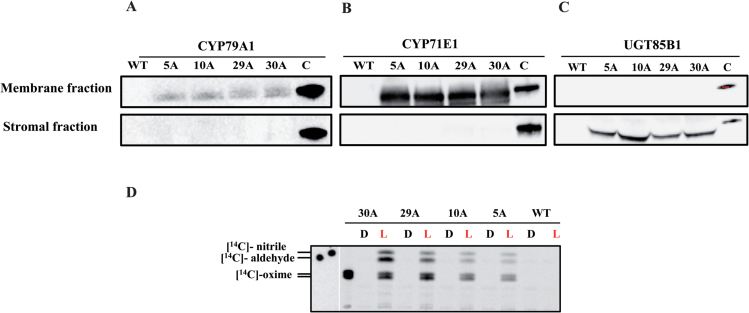
Expression of the three enzymes of the dhurrin operon in the chloroplast. Intact chloroplasts were fractionated into stromal and membrane fractions as indicated and separated using SDS-PAGE. Immunoblot analyses were carried out with primary antibodies against CYP79A1 (**A**), CYP71E1 (**B**), and UGT85B1 (**C**). As positive controls, purified His-tagged CYP79A1 and CYP71E1 proteins and GFP-tagged UGT85B1 were used. (**D**) *In vitro* light-driven activity assays performed on isolated thylakoids from the *DhuOp* transformed lines. The products produced from the [^14^C]-tyrosine substrate was analysed by TLC separation. Thylakoids from the wild-type were used as negative control. ‘L’ and ‘D’ denote incubation of the enzymatic assays in light or darkness. The assay mixtures were extracted with EtOAc and the organic phase applied to the TLC.

Western blot analysis using specific antibodies to CYP79A1, CYP71E1, and UGT85B1 demonstrated that all three enzymes were expressed from the *DhuOp* construct in the chloroplast, and the P450s were found to be enriched in thylakoid membranes of the transgenic lines (Fig. 2). The latter was demonstrated by preparation of intact chloroplasts from the transgenic *DhuOp* line and fractionation into a soluble stroma fraction and a membrane fraction. The two P450s were mainly localized in the membrane fraction (Fig. 2A, B) and UGT85B1 was found mainly in the soluble stroma fraction (Fig. 2C). At present, we cannot rule out that a fraction of the P450s also insert into the inner envelope of the chloroplast; however, either way the catalytic domain of the P450 will face the stroma. The expression of the two P450s from the stably integrated dhurrin operon yielded clearly detectable amounts of protein in the western blots. In a parallel approach we also introduced the genes encoding the two P450s through stable nuclear transformation (Supplementary methods, Fig. S2 and Table S1). The protein expression from these single gene constructs was too low to be detected by immunoblotting. This suggests that expression from chloroplast-integrated genes is higher and more robust compared to expression from nuclear integration.

The abundance of CYP79A1 and CYP71E1 enzymes in the thylakoids was estimated and compared to estimates of the abundance of the PsaD subunit of PSI (Supplementary Fig. S3). The amount of PsaD protein in wild-type *N. tabacum* was 3.37±0.64 mmol mol^−1^ chlorophyll, which is comparable to 2.16±0.24 mmol mol^−1^ chlorophyll reported previously in *A. thaliana* (Armbruster *et al.*, 2013). Hence by taking the PsaD protein as an indicator of how much PSI there is in the thylakoids, we found between 50- and 1000-fold less CYP79A1 and 2- to 5-fold less CYP71E1 enzyme compared to the abundance of PsaD in the *DhuOp* thylakoids (Table 1). Interestingly, the two P450 enzymes of the dhurrin pathway are apparently not present in equimolar concentrations, which might reflect different expression levels or stability.

**Table 1:** Abundance of PsaD subunit, and CYP79A1 and CYP71E1 enzymes in the thylakoids of the wild-type and *DhuOp* lines.

	PsaD (mmol/[mol Chl])	CYP79A1 (µmol/[mol Chl])	CYP71E1 (mmol/[mol Chl])
Wild type	3.37±0.64	0	0
5A	2.07±0.33	2.35±0.95	1.12±0.55
10A	2.38±0.19	12.59±1.84	0.59±0.20
29A	2.75±0.30	28.73±7.30	0.47±0.35
30A	2.61±0.42	35.73±8.53	0.61±0.42

To ascertain that the CYP79A1 and CYP71E1 proteins inserted into thylakoids were active, *in vitro* activity assays were carried out with the thylakoids. The light-driven conversion of L-[^14^C]-tyrosine to [^14^C]-*p*-hydroxyphenylacetaldoxime by the CYP79A1 enzyme and the subsequent conversion of [^14^C]-*p*-hydroxyphenylacetaldoxime to [^14^C]-*p*-hydroxyphenylacetonitrile and [^14^C]-*p*-hydroxybenzaldehyde by the CYP71E1 enzyme demonstrated that both P450s were functionally active in the thylakoids and were able to use photosynthetic reducing power instead of electron transfer mediated by POR to drive their catalytic reactions (Fig. 2D).

### The *in vivo* biosynthesis of dhurrin and other glucosides in the chloroplast

**Fig. 3.**
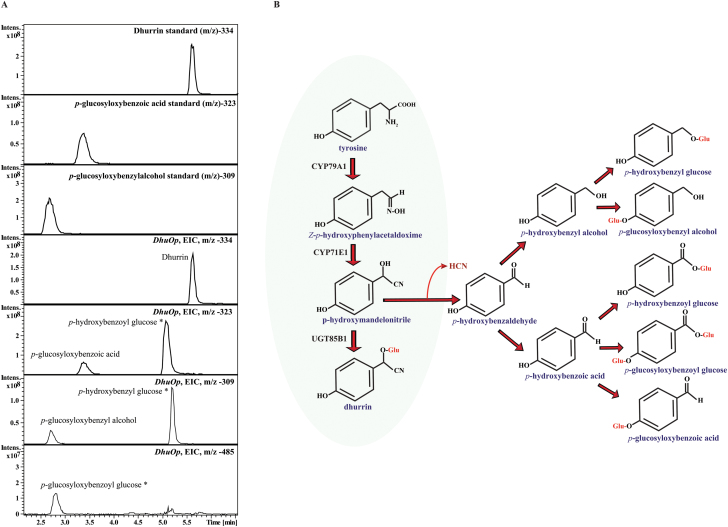
LC-MS based analysis of dhurrin and related glucosides produced by integration of the *DhuOp* operon construct in the chloroplast genome of *N. tabacum* and their predicted route of formation. (**A**) Extracted ion chromatograms of leaf extracts depicting the presence of glucosides (electron spray ionization mode). The production of dhurrin (m/z = 334.1) at retention time (Rt) 5.5min was demonstrated in the leaf extract obtained from the *DhuOp* line. In addition to dhurrin, additional glucosides derived from the intermediate *p*-hydroxybenzaldehyde were also identified. Standards of dhurrin, *p*-glucosyloxy-benzoic acid and *p*-glucosyloxy-benzylalcohol are shown. Identification of the metabolites indicated with an asterisk are based upon literature (Kristensen *et al.*, 2005). (**B**) Schematic representation of the dhurrin pathway illustrating the intermediates involved and other glucosylated products formed upon engineering the dhurrin pathway into the chloroplast of tobacco.

We next wanted to analyse whether the dhurrin pathway was active *in vivo* when relocated to the chloroplast and no exogenous substrates were applied. In theory the pathway should have access to both of its substrates, tyrosine and UDP-glucose, which are both produced in the chloroplast (Okazaki *et al.*, 2009; Rippert *et al.*, 2009). We therefore analysed the metabolite profile in the *DhuOp*-engineered *N. tabacum* lines by LC-MS. When the leaf extracts from the *DhuOp* lines were analysed, we observed the presence of dhurrin (m/z = 334.1) at the retention time (R_t_) 5.5min (Fig. 3A). In addition to dhurrin, other glucosides derived from *p*-hydroxybenzaldehyde (the dissociation product of *p*-hydroxymandelonitrile) were also identified: *p*-glucosyloxy-benzoic acid, *p*-glucosyloxy-benzoylglucose, *p*-glucosyloxy-benzylalcohol, *p*-hydroxybenzoylglucose, and *p*-hydroxybenzylglucose (Fig. 3A, B). The respective LC-MS mass fragmentation pattern of the dhurrin and other glucosides are shown in Supplementary Fig. S4.

### *In vivo* production of dhurrin corresponds to 0.2% of leaf dry weight

**Fig. 4.**
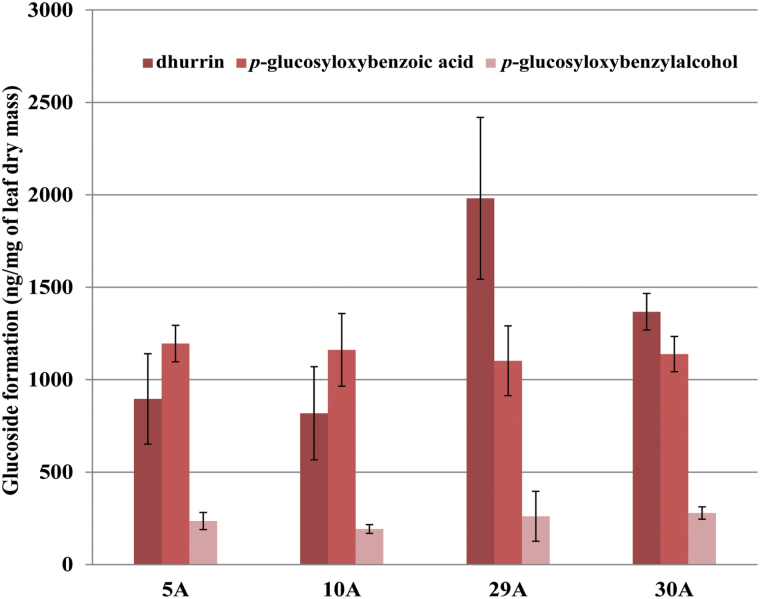
Quantification of dhurrin and other glucosides formed upon expression of the *DhuOp* operon construct in the chloroplast genome of *N. tabacum* using LC-MS. For quantification of the metabolites from leaf extracts, the third leaf from the top of 5-week-old tobacco plants was analysed. Three plants from each line were measured; the error bars indicate the standard deviation.

The amount of dhurrin and the turnover glucosides accumulating in the *DhuOp*-expressing *N. tabacum* leaves *in vivo* was quantified using LC-MS analysis based on the availability of authentic standards. The amount of dhurrin formed constituted between 0.1% and 0.2% of the leaf dry mass. In addition, the glucosides *p*-glucosyloxy-benzoic acid and *p*-glucosyloxy-benzylalcohol that are derived from *p*-hydroxybenzaldehyde constituted between 0.1% and 0.02% of leaf dry mass, respectively (Fig. 4). However, the other three glucosides formed could not be quantified to absolute amounts owing to the unavailability of chemically synthesized standards. Dhurrin and intermediates were produced both in young and old leaves (Supplementary Fig. S5), suggesting expression of the operon throughout plant development.

### Effect of expression of the *DhuOp* on the transgenic plants

**Fig. 5.**
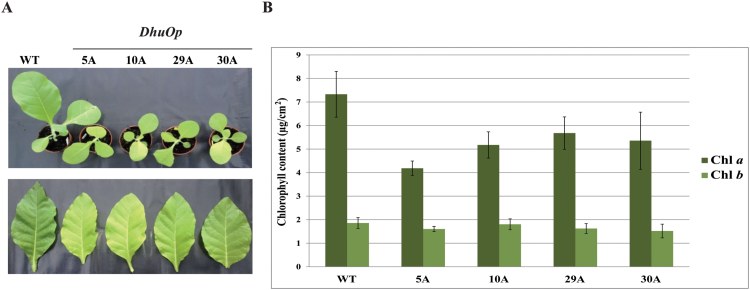
Phenotypic differences between wild-type *N. tabacum* and transplastomic lines expressing the *DhuOp* operon. (**A**) Visual differences seen between the wild-type and four transformed lines. Top: relative growth difference observed between 3-week-old wild-type plants and the *DhuOp* lines. Bottom: comparison of leaves harvested from 6-week-old wild-type and *DhuOp*-transformed *N. tabacum* lines. (**B**) The chlorophyll *a* (Chl *a*) and chlorophyll *b* (Chl *b*) content per leaf area in wild-type and *DhuOp* lines.

The *DhuOp* tobacco lines were fully fertile but displayed a clearly visible phenotype, with slower growth rate and paler leaves (Fig. 5A). The amount of chlorophyll *a* per area of the *DhuOp* lines was ~25–40% lower than that in the wild-type lines, whereas the chlorophyll *b* levels were less affected (Fig. 5B), suggesting a reduction in the photosystem cores and a smaller effect on the peripheral antenna.

**Fig. 6.**
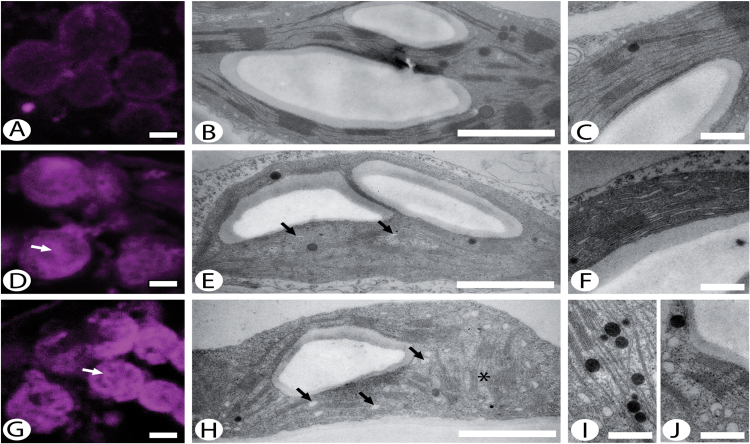
Chloroplast ultrastructure and staining of lipids in wild-type and selected *DhuOp* lines. (**A**, **D**, **G**) Single optical sections through Nile red-labelled cells showing lipids (in magenta) present in chloroplasts of the wild-type (A) and *DhuOp* lines 29A (D) and 5C (G). Note the strong, granular signal from the *DhuOP* lines (white arrows). (**B**, **C**, **E**, **F**, **H**, **I**, **J**) Ultrastructure of chloroplasts from the wild-type (B, C), *DhuOp* line 29A (E, F), and *DhuOp* line 5C (H–J) as observed by TEM. Membrane-bound vesicles appear from dilated thylakoid envelopes (black arrows). Also note the accumulation of plastoglobules (I) and the unusual thylakoid arrangement in 5C (asterisk in H). Scale bars: 2 µm (A, D, G), 1 µm (B, E, H), 0.35 µm (C, F, I, J).

In order to investigate the phenotype of the *DhuOp* lines in more detail, we visualized the distribution of lipids in the chloroplasts of selected *DhuOp* lines in comparison to the wild-type using Nile red staining in combination with confocal microscopy. Thylakoid ultrastructure was additionally analysed by TEM. A major difference between the wild-type and *DhuOp* lines was observed with regard to the distribution of lipids within the chloroplasts (Fig. 6). There was a greater quantity of presumed lipid droplets in *DhuOp* line 29A (Fig. 6D), and even more so in *DhuOp* line 5A (Fig. 6G), than in wild-type chloroplasts (Fig. 6A). Using xyz-stacks, it was confirmed that the lipid signal arose from within the chloroplasts (not shown) and that the lipid signal was higher from the *DhuOp* line chloroplasts using xyz-stacks (not shown).

The chloroplast structure in leaf tissues of wild-type and *DhuOp* lines was also examined using TEM. Dilation of the stroma thylakoids was evident in *DhuOp* line 29A (Fig. 6E, F), and line 5A displayed electron-dense plastoglobules (Fig. 6I) and numerous membrane-bound electron-translucent vesicles in the vicinity of thylakoid membranes (Fig. 6H, J). The clustered assemblies of lipids observed in *DhuOp* line 5A upon staining with Nile Red (Fig. 6G) could represent lipid droplets budding off from the envelope (Fig. 6J) and/or the plastoglobules (Fig. 6I), which are known to contain lipids (Kaup *et al.*, 2002). In *DhuOp* line 5A, a major fraction of the thylakoids were oriented perpendicular to the normal arrangement (Fig. 6H) while in the wild-type and *DhuOp* line 29A, the thylakoids surfaces ran parallel to the long axis of the chloroplast. Whether this is directly related to the expression of the dhurrin pathway or reflects slightly different developmental stages of the leaf tissues is currently unknown. There was a clear difference in the number of chloroplasts within the ultrathin sections between the *DhuOp* lines and the wild type. Roughly, the ratio between wild-type, line 29A, and line 5A chloroplasts was 10:6:3. While the chloroplasts from *DhuOp* line 29A were almost the size of those in the wild-type, *DhuOp* line 5A chloroplasts were clearly smaller than wild type, adding to the difference between them. With regard to the appearance of mitochondria and the number of starch grains within the chloroplasts, no differences were observed between wild-type and the *DhuOp* lines.

### Altered photosynthetic performance of the transplastomic lines

**Fig. 7.**
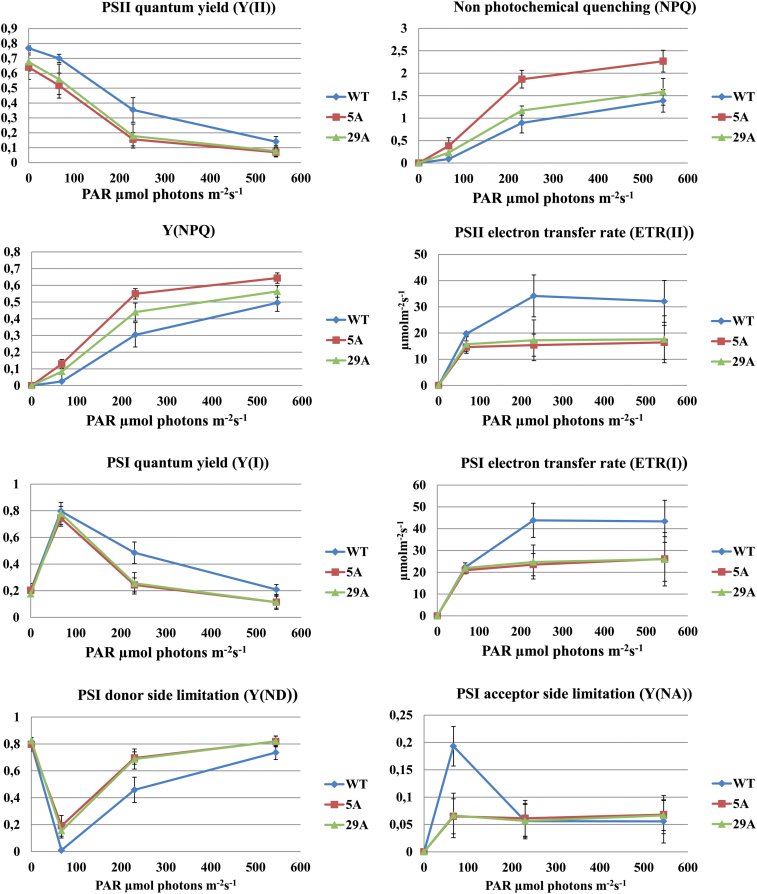
Characterization of photosynthetic performance in *N. tabacum* wild-type plants in comparison to *DhuOp*-expressing lines using DUAL-PAM measurements performed at different light intensities. All measurements were carried out using intact leaves (n = 3) of *N. tabacum*.

Further characterization of the photosynthetic apparatus and performance of the *DhuOp* lines was performed using DUAL-PAM fluorometer measurements on intact leaves of the wild-type and *DhuOp* 5A and 29A lines. Clear differences were observed. Maximum PSII efficiency in the dark (Fv/Fm) in *DhuOp* lines 5A and 29A was reduced in comparison to the wild type (Fig. 7A). A lower PSII efficiency was also seen under increasing light intensities. The PSI efficiency showed the same tendency under the respective light conditions (Fig. 7A).

In parallel to the lowered PSII and PSI efficiencies under increasing light intensities, the *DhuOp* lines showed enhanced non-photochemical quenching (NPQ), suggesting increased acidification of the lumen and induction of the xanthophyll cycle. In addition, a higher heat dissipation via the regulated photoprotective NPQ [Y(NPQ)] was also seen (Fig. 7A). The PSII and PSI electron transport rates were severely reduced in the two *DhuOp* lines, suggesting a restriction of electron flow at the donor or acceptor side of PSI. To distinguish between these possibilities, the quantum yield of non-photochemical energy dissipation in PSI due to donor side [Y(ND)] and acceptor side [Y(NA)] limitation was determined. The PSI donor side limitation Y(ND) values were significantly higher at lower light intensities for the *DhuOp* lines, indicating an enhanced build-up of the trans-thylakoid proton gradient under these conditions (Fig. 7A). This could either be caused by photosynthetic control at the cyt *b*_*6*_ *f* complex and/or down-regulation of PSII. However, at increasing light intensities, the PSI donor side limitation gradually reached the same values. By contrast, the Y(NA) values for the *DhuOp* lines were significantly lower (67 µmol photons m^−2^ s^−1^), whereafter Y(NA) values reached the same values as the wild type (Fig. 7A). In summary, the results indicate that the reduced photosynthetic fitness of the *DhuOp* lines resulted mainly from restricted electron flow to PSI or, alternatively, increased cyclic electron flow.

## Discussion

In this work, we successfully targeted the dhurrin pathway, which consists of two membrane-bound P450s and a soluble glucosyltransferase, to the chloroplasts of tobacco. The two P450s were found to be enriched in thylakoids and displayed light-dependent activity (Fig. 2D) when assayed *in vitro*. The activity *in vitro* was strictly light-driven, suggesting that the photo-reduced Fd was the direct electron donor for the P450s. Our study shows that these P450s, which in nature reside in the endoplasmic reticulum membranes of *S. bicolor*, can be stably expressed and assembled in the chloroplasts of tobacco and that they remain highly active when anchored to thylakoids (Fig. 2A, B). Relocation of the dhurrin pathway into tobacco chloroplasts eliminated the requirement for POR-mediated electron transfer to the P450s and circumvented the requirement for NADPH as the electron donor, two components that are crucial for the P450s to operate in the native environment of the endoplasmic reticulum of *S. bicolor* (Jensen *et al.*, 2011b). This finding is in accordance with results from previous *in vitro* reconstitution experiments (Jensen *et al.*, 2011a) and transient expression studies (Nielsen *et al.*, 2013), which showed that reduced Fd can replace POR and NADPH. The demonstration that this is also the case *in vivo* opens up a new avenue in metabolic engineering of P450s in which the NADPH-dependent native reductase is eliminated and replaced by photo-reduced Fd, which is abundant in chloroplasts (Cammack *et al.*, 1977; Hanke and Mulo, 2013). The availability of a potent reducing system poses a challenge when using hosts such as *E. coli* and *Saccharomyces cerevisiae* for expressing P450s. In the case of *S. cerevisiae*, co-expression of a plant POR together with the P450 is often necessary (Ro *et al.*, 2006; Paddon *et al.*, 2013). In *E. coli*, fusions of P450 and the respective POR have proven useful in this regard (Ajikumar *et al.*, 2010; Sadeghi and Gilardi, 2013).

In sorghum, it has been proposed that the UGT85B1 binds to CYP79A1 and CYP71E1 to form a metabolon, thus facilitating efficient channelling of the conversion of the tyrosine substrate into dhurrin (Nielsen *et al.*, 2008; Møller, 2010; Laursen *et al.*, 2015). The release of pathway intermediates and their derivatives was therefore examined in extracts from leaves of the *DhuOp*-expressing *N. tabacum* lines using LC-MS (Fig. 3). Apart from dhurrin, a number of glucosides derived from *p*-hydroxybenzaldehyde were found (Bak *et al.*, 2000). *p*-Hydroxybenzaldehyde is produced by dissociation of the labile cyanohydrin *p*-hydroxymandelonitrile, the product of the CYP71E1 enzyme (Sibbesen *et al.*, 1995; Kahn *et al.*, 1997; Bak *et al.*, 1998). The presence of numerous glucosides derived from *p*-hydroxybenzaldehyde in the leaf extracts of the *DhuOp*-expressing *N. tabacum* lines suggests that the coupling between the two P450s is efficient. This is also supported by the relative high abundance of CYP71E1 compared to the CYP79A1 (Table 1). However, the presence of turnover glucosides derived from *p*-hydroxybenzaldehyde, but not of any glucosides produced from *p*-hydroxyphenylacetaldoxime, in the leaf extracts suggests that *p*-hydroxymandelonitrile might not be efficiently channelled to the UGT85B1 enzyme for glucosylation. This could be interpreted as a lack of association between UGT85B1 and the two P450s as required for metabolon formation. The labile compound *p*-hydroxymandelonitrile would therefore not be stabilized by immediate glucosylation and, instead, dissociate into *p*-hydroxybenzaldehyde and hydrogen cyanide. Part of the *p*-hydroxybenzaldehyde formed would then be metabolized by non-specific dehydrogenases (Fig. 3B). These may then be glucosylated by UGT85B1 which has a broad substrate specificity and previously has been reported to catalyse glucosylation of, for example, *p*-hydroxybenzoic acid and *p*-hydroxyphenylethanol (Jones *et al.*, 1999). Alternatively, *p*-hydroxybenzaldehyde may diffuse into the cytosol and be metabolized and subsequently glucosylated by other UGTs present in the cytosol. It should be noted that the formation of *p*-hydroxybenzaldehyde is accompanied by the release of stoichiometric amounts of toxic hydrogen cyanide that if not further metabolized may contribute to the slower growth rate of the *DhuOp* lines, for example, by inhibiting complex IV in the mitochondrial electron transport chain. Surprisingly, neither the intermediates (*E*)- and (*Z*)-*p*-hydroxyphenylacetaldoxime nor any glucosides derived from *p*-hydroxyphenylacetaldoxime were detected, whereas such constituents were found upon expression of the dhurrin pathway genes in *A. thaliana* and tobacco where the pathway was not targeted to the chloroplast (Bak *et al.*, 2000). This would imply that CYP79A1 and CYP71E1 are able to form a complex when present in the membrane of the endoplasmic reticulum as well as in the thylakoids.

Chloroplasts are natural light-driven cell factories that, in nature, synthesize a number of key precursors for the biosynthesis of terpenoids (Vranova *et al.*, 2013) and aromatic amino acids (Tzin and Galili, 2010; Maeda and Dudareva, 2012). In plants, the biosynthesis of tyrosine takes place in the chloroplast from the substrate chorismate via prephenate and arogenate (Tzin and Galili, 2010). Several studies have shown that the genes encoding the enzymes catalysing synthesis of aromatic amino acids are up-regulated as a response to wounding and pathogen infection (Maeda and Dudareva, 2012). Relocation of the dhurrin pathway to the chloroplast, therefore, benefits from the endogenous production and availability of tyrosine for the biosynthesis of dhurrin. In the present work, despite the relocation of the entire pathway to the chloroplast and constitutive expression of the enzymes, we observed dhurrin accumulation of 0.1–0.2% of plant dry matter. In a previous study in *A. thaliana,* where the dhurrin pathway was engineered for nuclear expression and endoplasmic reticulum localization, the amount of dhurrin accumulated was 4% of plant dry matter (Kristensen *et al.*, 2005). Even though up-regulating tyrosine biosynthesis upon demand is feasible in the chloroplasts, the abundance and availability of the activated sugar donor UDP-glucose in the chloroplast is presently unclear. UDP-glucose has been demonstrated to be present in chloroplasts of *A. thaliana* and is required for the biosynthesis of the sulfolipid sulfoquinovosyldiacylglycerol (Okazaki *et al.*, 2009). Hence, the promiscuity of UGT85B1 may result in a depletion of the UDP-glucose pool in *DhuOp* lines and constitute a bottleneck in dhurrin production.

The *DhuOp*-expressing *N. tabacum* lines were fertile but displayed a clear phenotype, with paler green leaves, a slower growth rate, and delayed flowering. In addition, the engineered lines showed an altered thylakoid ultrastructure as seen by TEM. These observations coincide with significant changes in photosynthetic parameters in the *DhuOp* lines (Fig. 7A). Although the precise reason(s) for these negative effects in the *DhuOp* lines remains unclear, it seems reasonable to speculate that spatial competition within the already protein-crowded thylakoids could contribute to the phenotype of the *DhuOp* lines. This might affect the optimal lateral heterogeneity of the PSI and PSII complexes within the thylakoid membrane, and consequently affect the protein dynamics within the thylakoids that are required for optimal photosynthetic efficiency. Direct competition for the photosynthetic reducing power between the production of activated sugars and dhurrin, and other essential processes such as protein synthesis (including PSII repair) and energy metabolism also cannot be excluded (Shachar-Hill, 2013; Farré *et al.*, 2014).

## Conclusions

In this work, we established stable lines of tobacco expressing the dhurrin pathway in the chloroplast. We demonstrated that the P450s usually residing in the endoplasmic reticulum membranes can be stably expressed in the chloroplast and remain highly active when localized to the thylakoids. Furthermore, we also showed that the two P450s of the pathway can be light-driven, receiving electrons directly from photo-reduced Fd. Thus, our concept eliminates the need for a P450 reductase and NADPH for driving P450 catalysis. This opens up novel opportunities to use chloroplasts as a platform for expressing P450s that are involved in biosynthetic pathways leading to high-value compounds.

## Supplementary data

Supplementary data are available at *JXB* online.

Supplementary materials and methods. Expression of the individual CYP79A1 and CYP71E1 enzymes and their targeting to the thylakoids of genetically transformed *N. benthamiana.*

Table S1. Details of primer pairs used for confirming the integration of *Fd(TP)-CYP79A1* and *Fd(TP)-CYP71E1* gene constructs in the nuclear genome.

Fig. S1. Seed tests to assess homoplasmy of transplastomic lines.

Fig. S2. Nuclear transformation of *N. benthamiana* with the genes encoding the two P450s involved in the dhurrin pathway.

Fig. S3. The quantification of (A) PsaD subunit (B) CYP79A1 and (C) CYP71E1 in the thylakoids of wild-type and *DhuOp* lines.

Fig. S4. Mass fragmentation pattern for dhurrin and selected turnover glucosides produced from *p*-hydroxybenzaldehyde detected using LC-MS.

Fig. S5. LC-MS analysis of dhurrin and glucosides on the extracts from leaves of different age from the *DhuOp* line 5A.

Supplementary Data

## References

[CIT0001] Ajikumar PK Xiao WH Tyo KE Wang Y Simeon F Leonard E Mucha O Phon TH Pfeifer B Stephanopoulos G 2010 Isoprenoid pathway optimization for Taxol precursor overproduction in *Escherichia coli* . Science 330, 70–74. 20929806 10.1126/science.1191652 PMC3034138

[CIT0002] Armbruster U Labs M Pribil M Viola S Xu W Scharfenberg M Hertle AP Rojahn U Jensen PE Rappaport F Joliot P Dörmann P Wanner G Leister D 2013 Arabidopsis CURVATURE THYLAKOID1 proteins modify thylakoid architecture by inducing membrane curvature. The Plant Cell 25, 2661–2678. 23839788 10.1105/tpc.113.113118 PMC3753390

[CIT0003] Aslan S Sun C Leonova S Dutta P Dormann P Domergue F Stymne S Hofvander P 2014 Wax esters of different compositions produced via engineering of leaf chloroplast metabolism in *Nicotiana benthamiana* . Metabolic Engineering 25, 103–112. 25038447 10.1016/j.ymben.2014.07.001

[CIT0004] Bach SS Bassard JE Andersen-Ranberg J Moldrup ME Simonsen HT Hamberger B 2014 High-throughput testing of terpenoid biosynthesis candidate genes using transient expression in *Nicotiana benthamiana* . Methods in Molecular Biology 1153, 245–255. 24777803 10.1007/978-1-4939-0606-2_18

[CIT0005] Bak S Kahn R Nielsen H Møller B Halkier B 1998 Cloning of three A-type cytochromes P450, CYP71E1, CYP98, and CYP99 from *Sorghum bicolor* (L.) Moench by a PCR approach and identification by expression in *Escherichia coli* of CYP71E1 as a multifunctional cytochrome P450 in the biosynthesis of the cyanogenic glucoside dhurrin. Plant Molecular Biology 36, 393–405. 9484480 10.1023/a:1005915507497

[CIT0006] Bak S Olsen CE Halkier BA Møller BL 2000 Transgenic tobacco and Arabidopsis plants expressing the two multifunctional sorghum cytochrome P450 enzymes, CYP79A1 and CYP71E1, are cyanogenic and accumulate metabolites derived from intermediates in dhurrin biosynthesis. Plant Physiology 123, 1437–1448. 10938360 10.1104/pp.123.4.1437 PMC59100

[CIT0007] Baker NR 2008 Chlorophyll fluorescence: a probe of photosynthesis in vivo. Annual Review of Plant Biology 59, 89–113. 10.1146/annurev.arplant.59.032607.092759 18444897

[CIT0008] Bock R 2001 Transgenic plastids in basic research and plant biotechnology. Journal of Molecular Biology 312, 425–438. 11563907 10.1006/jmbi.2001.4960

[CIT0009] Bock R 2014 Genetic engineering of the chloroplast: novel tools and new applications. Current Opinion in Biotechnology 26, 7–13. 24679252 10.1016/j.copbio.2013.06.004

[CIT0010] Bock R 2015 engineering plastid genomes: methods, tools, and applications in basic research and biotechnology. Annual Review of Plant Biology 66, 211–241. 10.1146/annurev-arplant-050213-040212 25494465

[CIT0011] Bock R Warzecha H 2010 Solar-powered factories for new vaccines and antibiotics. Trends in Biotechnology 28, 246–252. 20207435 10.1016/j.tibtech.2010.01.006

[CIT0012] Cammack R Rao KK Bargeron CP Hutson KG Andrew PW Rogers LJ 1977 Midpoint redox potentials of plant and algal ferredoxins. Biochemical Journal 168, 205–209. 202262 10.1042/bj1680205 PMC1183753

[CIT0013] Doyle JJ Doyle J.L 1990 Isolation of plant DNA from fresh tissue. Focus 12, 13–15.

[CIT0014] Elghabi Z Ruf S Bock R 2011 Biolistic co-transformation of the nuclear and plastid genomes. The Plant Journal 67, 941–948. 21554457 10.1111/j.1365-313X.2011.04631.x

[CIT0015] Emadpour M Karcher D Bock R 2015 Boosting riboswitch efficiency by RNA amplification. Nucleic Acids Research 43, e66. 25824954 10.1093/nar/gkv165 PMC4446413

[CIT0016] Farré G Blancquaert D Capell T Straeten DVD Christou P Zhu C 2014 Engineering complex metabolic pathways in plants. Annual Review of Plant Biology 65, 187–223. 10.1146/annurev-arplant-050213-035825 24579989

[CIT0017] Gnanasekaran T Vavitsas K Andersen-Ranberg J Nielsen AZ Olsen CE Hamberger B Jensen PE 2015 Heterologous expression of the isopimaric acid pathway in *Nicotiana benthamiana* and the effect of N-terminal modifications of the involved cytochrome P450 enzyme. Journal of Biological Engineering 9, 24. 26702299 10.1186/s13036-015-0022-z PMC4688937

[CIT0018] Hager M Biehler K Illerhaus J Ruf S Bock R 1999 Targeted inactivation of the smallest plastid genome‐encoded open reading frame reveals a novel and essential subunit of the cytochrome b6f complex. EMBO Journal 18, 5834–5842. 10545095 10.1093/emboj/18.21.5834 PMC1171649

[CIT0019] Hanke GUY Mulo P 2013 Plant type ferredoxins and ferredoxin-dependent metabolism. Plant, Cell and Environment 36, 1071–1084. 10.1111/pce.12046 23190083

[CIT0020] Hannemann F Bichet A Ewen KM Bernhardt R 2007 Cytochrome P450 systems--biological variations of electron transport chains. Biochemica et Biophysica Acta 1770, 330–344. 10.1016/j.bbagen.2006.07.017 16978787

[CIT0021] Jensen K Jensen PE Moller BL 2011a. Light-driven cytochrome p450 hydroxylations. ACS Chemical Biology 6, 533–539. 21323388 10.1021/cb100393j

[CIT0022] Jensen K Moller BL 2010 Plant NADPH-cytochrome P450 oxidoreductases. Phytochemistry 71, 132–141. 19931102 10.1016/j.phytochem.2009.10.017

[CIT0023] Jensen K Osmani SA Hamann T Naur P Moller BL 2011b. Homology modeling of the three membrane proteins of the dhurrin metabolon: catalytic sites, membrane surface association and protein-protein interactions. Phytochemistry 72, 2113–2123. 21620426 10.1016/j.phytochem.2011.05.001

[CIT0024] Jones PR Møller BL Høj PB 1999 The UDP-glucose:p-hydroxymandelonitrile-O-glucosyltransferase that catalyses the last step in synthesis of the cyanogenic glucoside dhurrin in *Sorghum bicolor*. Isolation, cloning, heterologous expression, and substrate specificity. Journal of Biological Chemistry 274, 35483–35491. 10585420 10.1074/jbc.274.50.35483

[CIT0025] Kahn RA Bak S Svendsen I Halkier BA Moller BL 1997 Isolation and reconstitution of cytochrome P450ox and in vitro reconstitution of the entire biosynthetic pathway of the cyanogenic glucoside dhurrin from sorghum. Plant Physiology 115, 1661–1670. 9414567 10.1104/pp.115.4.1661 PMC158632

[CIT0026] Kaup MT Froese CD Thompson JE 2002 A role for diacylglycerol acyltransferase during leaf senescence. Plant Physiology 129, 1616–1626. 12177474 10.1104/pp.003087 PMC166749

[CIT0027] Krech K Fu HY Thiele W Ruf S Schottler MA Bock R 2013 Reverse genetics in complex multigene operons by co-transformation of the plastid genome and its application to the open reading frame previously designated psbN. The Plant Journal 75, 1062–1074. 23738654 10.1111/tpj.12256

[CIT0028] Kristensen C Morant M Olsen CE Ekstrom CT Galbraith DW Moller BL Bak S 2005 Metabolic engineering of dhurrin in transgenic Arabidopsis plants with marginal inadvertent effects on the metabolome and transcriptome. Proceedings of the National Academy of Sciences of the United States of America 102, 1779–1784. 15665094 10.1073/pnas.0409233102 PMC545087

[CIT0029] Lassen LM Nielsen AZ Ziersen B Gnanasekaran T Møller BL Jensen PE 2014 Redirecting photosynthetic electron flow into light-driven synthesis of alternative products including high-value bioactive natural compounds. ACS Synthetic Biology 3, 1–12. 24328185 10.1021/sb400136f

[CIT0030] Laursen T Moller BL Bassard JE 2015 Plasticity of specialized metabolism as mediated by dynamic metabolons. Trends in Plant Science 20, 20–32. 25435320 10.1016/j.tplants.2014.11.002

[CIT0031] Li W Ruf S Bock R 2010 Chloramphenicol acetyltransferase as selectable marker for plastid transformation. Plant Molecular Biology 76, 443–451. 20721602 10.1007/s11103-010-9678-4

[CIT0032] Lichtenthaler HK 2007 Biosynthesis, accumulation and emission of carotenoids, alpha-tocopherol, plastoquinone, and isoprene in leaves under high photosynthetic irradiance. Photosynthesis Research 92, 163–179. 17634750 10.1007/s11120-007-9204-y

[CIT0033] Lu Y Rijzaani H Karcher D Ruf S Bock R 2013 Efficient metabolic pathway engineering in transgenic tobacco and tomato plastids with synthetic multigene operons. Proceedings of the National Academy of Sciences of the United States of America 110, E623–632. 23382222 10.1073/pnas.1216898110 PMC3581966

[CIT0034] Maeda H Dudareva N 2012 The shikimate pathway and aromatic amino acid biosynthesis in plants. Annual Review of Plant Biology 63, 73–105. 10.1146/annurev-arplant-042811-105439 22554242

[CIT0035] Maliga P 2004 Plastid transformation in higher plants. Annual Review of Plant Biology 55, 289–313. 10.1146/annurev.arplant.55.031903.141633 15377222

[CIT0036] Maxwell K Johnson GN 2000 Chlorophyll fluorescence—a practical guide. Journal of Experimental Botany 51, 659–668. 10938857 10.1093/jxb/51.345.659

[CIT0037] Møller B Seigler D 1999 Biosynthesis of cyanogenic glucosides, cyanolipids and related compounds. Plant amino acids, biochemistry and biotechnology . Marcel Dekker, New York, 563–609.

[CIT0038] Møller BL 2010 Dynamic metabolons. Science 330, 1328–1329. 21127236 10.1126/science.1194971

[CIT0039] Nielsen AZ Ziersen B Jensen K Lassen LM Olsen CE Møller BL Jensen PE 2013 Redirecting photosynthetic reducing power toward bioactive natural product synthesis. ACS Synthetic Biology 2, 308–315. 23654276 10.1021/sb300128r

[CIT0040] Nielsen KA Tattersall DB Jones PR Moller BL 2008 Metabolon formation in dhurrin biosynthesis. Phytochemistry 69, 88–98. 17706731 10.1016/j.phytochem.2007.06.033

[CIT0041] Okazaki Y Shimojima M Sawada Y Toyooka K Narisawa T Mochida K Tanaka H Matsuda F Hirai A Hirai MY Ohta H Saito K 2009 A chloroplastic UDP-glucose pyrophosphorylase from Arabidopsis is the committed enzyme for the first step of sulfolipid biosynthesis. The Plant Cell 21, 892–909. 19286968 10.1105/tpc.108.063925 PMC2671695

[CIT0042] Paddon CJ Westfall PJ Pitera DJ Benjamin K Fisher K McPhee D Leavell MD Tai A Main A Eng D Polichuk DR Teoh KH Reed DW Treynor T Lenihan J Fleck M Bajad S Dang G Dengrove D Diola D Dorin G Ellens KW Fickes S Galazzo J Gaucher SP Geistlinger T Henry R Hepp M Horning T Iqbal T Jiang H Kizer L Lieu B Melis D Moss N Regentin R Secrest S Tsuruta H Vazquez R Westblade LF Xu L Yu M Zhang Y Zhao L Lievense J Covello PS Keasling JD Reiling KK Renninger NS Newman JD 2013 High-level semi-synthetic production of the potent antimalarial artemisinin. Nature 496, 528–532. 23575629 10.1038/nature12051

[CIT0043] Rippert P Puyaubert J Grisollet D Derrier L Matringe M 2009 Tyrosine and phenylalanine are synthesized within the plastids in Arabidopsis. Plant Physiology 149, 1251–1260. 19136569 10.1104/pp.108.130070 PMC2649395

[CIT0044] Ro DK Paradise EM Ouellet M Fisher KJ Newman KL Ndungu JM Ho KA Eachus RA Ham TS Kirby J Chang MC Withers ST Shiba Y Sarpong R Keasling JD 2006 Production of the antimalarial drug precursor artemisinic acid in engineered yeast. Nature 440, 940–943. 16612385 10.1038/nature04640

[CIT0045] Sadeghi SJ Gilardi G 2013 Chimeric P450 enzymes: activity of artificial redox fusions driven by different reductases for biotechnological applications. Biotechnology and Applied Biochemistry 60, 102–110. 23586997 10.1002/bab.1086

[CIT0046] Sanford JC 1990 Biolistic plant transformation. Physiologia Plantarum 79, 206–209.

[CIT0047] Schlichting I 2000 The catalytic pathway of cytochrome P450cam at atomic resolution. Science 287, 1615–1622. 10698731 10.1126/science.287.5458.1615

[CIT0048] Shachar-Hill Y 2013 Metabolic network flux analysis for engineering plant systems. Current Opinion in Biotechnology 24, 247–255. 23395406 10.1016/j.copbio.2013.01.004

[CIT0049] Shinozaki OM Tanaka M Wakasugi T Hayashida N Matsubayashi T Zaita N Chunwongse J Obokata J Yamaguchi-Shinozaki K Ohto C Torazawa K Meng BY Sugita M Deno H Kamogashira T Yamada K Kusuda J Takaiwa F Kato A Tohdoh N Shimada H Sugiura M 1986 The complete nucleotide sequence of the tobacco chloroplast genome: its gene organization and expression. EMBO Journal 5, 2043–2049. 16453699 10.1002/j.1460-2075.1986.tb04464.x PMC1167080

[CIT0050] Sibbesen O Koch B Halkier BA Møller BL 1995 Cytochrome P-450 is a multifunctional heme-thiolate enzyme catalyzing the conversion of l-tyrosine to p-hydroxyphenylacetaldehyde oxime in the biosynthesis of the cyanogenic glucoside dhurrin in *Sorghum bicolor* (L.) Moench. Journal of Biological Chemistry 270, 3506–3511. 7876084 10.1074/jbc.270.8.3506

[CIT0051] Sparkes IA Runions J Kearns A Hawes C 2006 Rapid, transient expression of fluorescent fusion proteins in tobacco plants and generation of stably transformed plants. Nature Protocols 1, 2019–2025. 17487191 10.1038/nprot.2006.286

[CIT0052] Takos A Lai D Mikkelsen L Abou Hachem M Shelton D Motawia MS Olsen CE Wang TL Martin C Rook F 2010 Genetic screening identifies cyanogenesis-deficient mutants of *Lotus japonicus* and reveals enzymatic specificity in hydroxynitrile glucoside metabolism. The Plant Cell 22, 1605–1619. 20453117 10.1105/tpc.109.073502 PMC2899875

[CIT0053] Tattersall DB Bak S Jones PR Olsen CE Nielsen JK Hansen ML Hoj PB Moller BL 2001 Resistance to an herbivore through engineered cyanogenic glucoside synthesis. Science 293, 1826–1828. 11474068 10.1126/science.1062249

[CIT0054] Tzin V Galili G 2010 New insights into the shikimate and aromatic amino acids biosynthesis pathways in plants. Molecular Plant 3, 956–972. 20817774 10.1093/mp/ssq048

[CIT0055] Verhounig A Karcher D Bock R 2010 Inducible gene expression from the plastid genome by a synthetic riboswitch. Proceedings of the National Academy of Sciences of the United States of America 107, 6204–6209. 20308585 10.1073/pnas.0914423107 PMC2852001

[CIT0056] Vranova E Coman D Gruissem W 2013 Network analysis of the MVA and MEP pathways for isoprenoid synthesis. Annual Review of Plant Biology 64, 665–700. 10.1146/annurev-arplant-050312-120116 23451776

[CIT0057] Werck-Reichhart D Feyereisen R 2000 Cytochromes P450: a success story. Genome Biology 1, reviews3003.3001–reviews3003.3009. 11178272 10.1186/gb-2000-1-6-reviews3003 PMC138896

[CIT0058] Zhou F Badillo-Corona JA Karcher D Gonzalez-Rabade N Piepenburg K Borchers AMI Maloney AP Kavanagh TA Gray JC Bock R 2008 High-level expression of human immunodeficiency virus antigens from the tobacco and tomato plastid genomes. Plant Biotechnology Journal 6, 897–913. 19548344 10.1111/j.1467-7652.2008.00356.x

[CIT0059] Zhou F Karcher D Bock R 2007 Identification of a plastid intercistronic expression element (IEE) facilitating the expression of stable translatable monocistronic mRNAs from operons. The Plant Journal 52, 961–972. 17825052 10.1111/j.1365-313X.2007.03261.x PMC2230500

