# Supplementary material for: Transfer of the cytochrome P450-dependent dhurrin pathway from Sorghum bicolor into Nicotiana tabacum chloroplasts for light-driven synthesis
Source: J Exp Bot. 2016 Mar 11;67(8):2495–506. doi: 10.1093/jxb/erw067 (PMC4809297; doi:10.1093/jxb/erw067)
Supplement: Supplementary Data [file supp_67_8_2495__index.html]

Transfer of the cytochrome P450-dependent dhurrin pathway from Sorghum bicolor into Nicotiana tabacum chloroplasts for light-driven synthesis — Transfer of the cytochrome P450-dependent dhurrin pathway from Sorghum bicolor into Nicotiana tabacum chloroplasts for light-driven synthesis — Supplementary Data 

# Transfer of the cytochrome P450-dependent dhurrin pathway from *Sorghum bicolor* into *Nicotiana tabacum* chloroplasts for light-driven synthesis

## Supplementary Data

Data files

- supplementary\_methods\_table\_S1\_figures\_S1\_S5.pdf - Supplementary Data
