# Supplementary material for: Transfer of the cytochrome P450-dependent dhurrin pathway from Sorghum bicolor into Nicotiana tabacum chloroplasts for light-driven synthesis
Source: J Exp Bot. 2016 Mar 11;67(8):2495–506. doi: 10.1093/jxb/erw067 (PMC4809297; doi:10.1093/jxb/erw067)
Supplement: Supplementary Data [file supp_erw067_supplementary_methods_table_S1_figures_S1_S5.pdf]

Supplementary materials and methods

Transfer of the cytochrome P450-dependent dhurrin pathway from *Sorghum bicolor* into tobacco chloroplasts for light-driven synthesis

Thiyagarajan Gnanasekaran, Daniel Karcher, Agnieszka Zygadlo Nielsen, Helle Juel Martens, Stephanie Ruf, Xenia Kroop, Carl Erik Olsen, Mohammed Saddik Motawie, Mathias Pribil, Birger Lindberg Møller, Ralph Bock and Poul Erik Jensen

Expression of the individual CYP79A1 and CYP71E1 enzymes and their targeting to the thylakoids of genetically transformed *N. benthamiana*

Materials and methods

Plant material and growth conditions

Tobacco (*N. benthamiana*) plants were grown under greenhouse conditions (16 h light/8 h dark cycle; 25°C/20°C; light intensity of 200–600 µmol m^{-2} s^{-1}).

Cloning, vector construction and transformation of *A. tumefaciens*

Single constructs harboring the coding region of the *CYP79A1* or *CYP71E1* gene fused to the ferredoxin transit peptide (*Fd(TP)*) were built (Nielsen *et al.*, 2013) and incorporated into the pEAQ-HT plasmid (Sainsbury *et al.*, 2009). Electrocompetent *A. tumefacians* (LBA4404) cells were prepared (Dulk-Ras and Hooykaas, 1995) and transformed with the vector pEAQ-HT harboring the *Fd(TP)-CYP79A1* and *Fd(TP)-CYP71E1* constructs by electroporation (400 ohm resistance, 2.5 kV, 25 µF capacitance; Gene Pulser, Biorad). The transformed cells were plated on YEP plates with 50 mg/l kanamycin and 50 mg/l rifampicin for selection.

Nuclear transformation of *N. benthamiana*

The *A.tumefacians* transformants containing the vector pEAQ-HT carrying *Fd (TP)-CYP79A1* and *Fd(TP)-CYP71E1* were grown for 48-72 h at 28°C in YEP media supplied with 25 mg/l rifampicin and 50 mg/l kanamycin. Nuclear transformation of *N. benthamiana* was carried out (Horsch RB, 1985).

Seed sterilization and regeneration of nuclear transformed lines

Seeds obtained from nuclear transformed *N. benthamiana* lines were surface sterilized (Fischer and Hain, 1995), placed on plates with MS medium containing 50 mg/l kanamycin, and germination was initiated by incubation in the dark for 48 h at 4°C followed by a 2 h incubation in the light (100-200 µmol $m^{-2}s^{-1}$) at room temperature. The plates were then incubated at room temperature in the dark for 2 d and kept in the growth chamber with a light intensity of 200 µmol m^{-2} s^{-1} at 25°C.

Genomic DNA purification and PCR

Genomic DNA was isolated from putative nuclear *N. benthamiana* transformants(Edwards *et al.*, 1991). To confirm successful transformation, 1 µL of the genomic DNA was used as a template for PCR using the primer pairs 79-F and 79-R for *Fd(TP)-CYP79A1* transgenic lines and 71-F and 71-R for *Fd(TP)-CYP71E1* transgenic lines (Supplementary Table S1).

Results

Each individual P450 of the dhurrin pathway was targeted to the chloroplast of *N. benthamiana* by fusing the N-terminus of the protein to the first 52 amino acids of the transit peptide of the *Arabidopsis thaliana* ferredoxin protein FedA (NCBI gene ID: 22136515; *Fd(TP);* Fig. S2A). Using the pEAQ-HT expression vector, the two fusion constructs (*Fd(TP)-CYP79A1* and *Fd(TP)-CYP71E1*) were transformed into the nuclear genome of *N. benthamiana*. For each construct, 3-5 transformed lines were obtained from two independent transformation events and analyzed. Genomic DNA was extracted and successful integration of the individual genes in the nuclear genome of the plants was verified by PCR. The size of the PCR product obtained for the *CYP79A1* and *CYP71E1* lines were 190 bp and 545 bp, respectively, which was in accordance with the expected size (Fig. S2B).

In vitro activity assays were performed with thylakoids prepared from the transgenic lines to assess the functionality of the incorporated P450s. The *in vitro* activity assays were carried out using radiolabeled substrates allowing a sensitive detection of enzymatic activity (Jensen *et al.*, 2011; Nielsen *et al.*, 2013). The thylakoids were solubilized with *n*-dodecyl-*β*-D-maltoside (*β*-DM) and incubated with exogenous ferredoxin (Fd) and the radiolabeled substrates [^{14}C]-tyrosine for CYP79A1 thylakoids or [^{14}C]-*p*-hydroxyphenylacetaldoxime for CYP71E1 thylakoids. The reaction mixtures were incubated in both darkness and light (Fig. S2C). The presence of *de novo* synthesized radioactively labelled products in the light-incubated samples clearly demonstrates that both P450s are present and active in the thylakoids of the transgenic plants. In addition, the products are only formed in the light thus demonstrating the requirement for light-driven electron transport to the P450s for these to catalyze the metabolic conversions, i.e. [^{14}C]-*p*-hydroxyphenylacetaldoxime formation by the CYP79A1 thylakoids,

and [^{14}C]-*p*-hydroxyphenylacetonitrile and [^{14}C]-*p*-hydroxybenzaldehyde formation by the CYP71E1 thylakoids. The CYP79A1 and CYP71E1-containing thylakoid preparations were probed with specific antibodies for CYP79A1 and CYP71E1. The immunoblot analyses did not reveal detectable expression of either CYP79A1 or CYP71E1 demonstrating that the P450s are present at very low levels in the thylakoids of the stably transformed lines.

References

Dulk-Ras A, Hooykaas PJ. 1995. Electroporation of *Agrobacterium tumefaciens*. In: Nickoloff J, ed. Plant Cell Electroporation and Electrofusion Protocols, Vol. 55: Springer New York, 63-72.

Edwards K, Johnstone C, Thompson C. 1991. A simple and rapid method for the preparation of plant genomic DNA for PCR analysis. Nucleic Acids Research **19**, 1349.

Fischer R, Hain R. 1995. Chapter 29 Tobacco protoplast transformation and use for functional analysis of newly isolated genes and gene constructs. Methods Cell Biology **50**, 401-410.

Horsch RB FJ, Hoffmann NL, Rogers SG and Fraley RT. 1985. A simple and general method for transferring genes into plants. Science **227**, 1229-1231.

Jensen K, Jensen PE, Moller BL. 2011. Light-driven cytochrome P450 hydroxylations. ACS Chemical Biology **6**, 533-539.

Nielsen AZ, Ziersen B, Jensen K, Lassen LM, Olsen CE, Møller BL, Jensen PE. 2013. Redirecting photosynthetic reducing power toward bioactive natural product synthesis. ACS Synthetic Biology **2**, 308-315.

Sainsbury F, Thuenemann EC, Lomonossoff GP. 2009. pEAQ: versatile expression vectors for easy and quick transient expression of heterologous proteins in plants. Plant Biotechnology Journal **7**, 682-693.

Table S1: Details of primer pairs used for confirming the integration of *Fd(TP)-CYP79A1* and *Fd(TP)-CYP71E1* gene constructs in the nuclear genome.

Forward primer	Reverse primer	Comments
**79-F**<br>CTCCGCGATCCTCAAACTGC	**79-R**<br>ATCTCCGGCAGGTTGCCGA	Primer pair used for confirming *Fd-CYP79A1* transgene integration into the nucleus of *N. benthamiana*
**71-F**<br>CGCACTTCGAGCTCATACCG	**71-R**<br>ATCGCAAGACCGGCAACAG	Primer pair used for confirming *Fd-CYP79A1* transgene integration into the nucleus of *N. benthamiana*

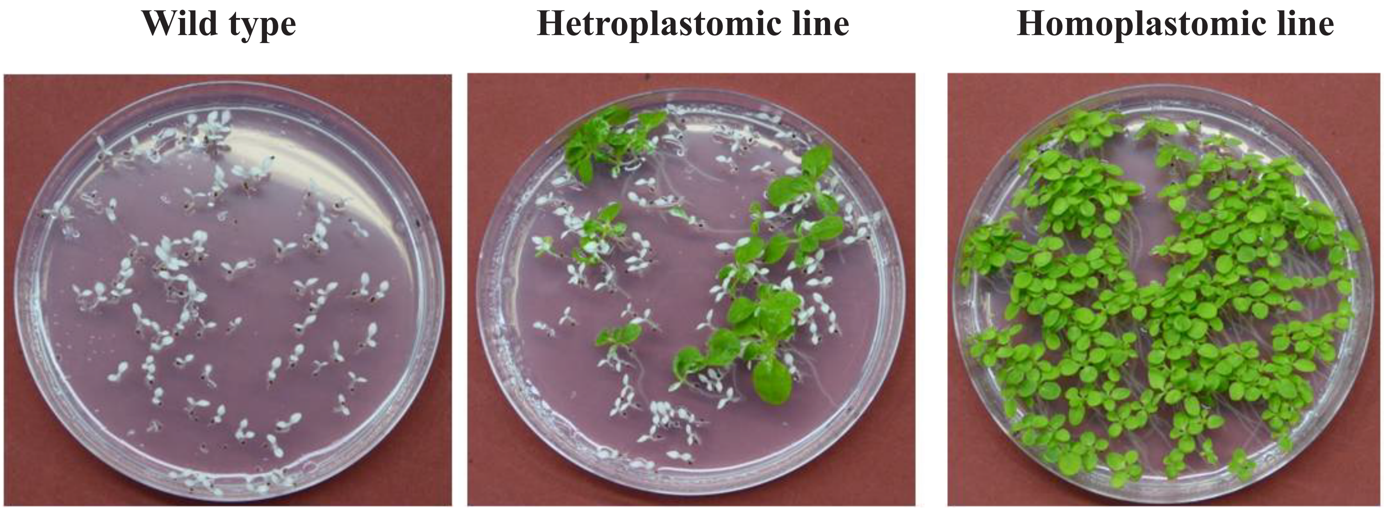

Fig. S1. Seed tests to assess homoplasmy of transplastomic lines. Seeds from a wild-type plant (left), a hetero-plastomic plant (center) and a homoplastomic plant (right) were germinated on synthetic medium containing 500 mg/L spectinomycin.

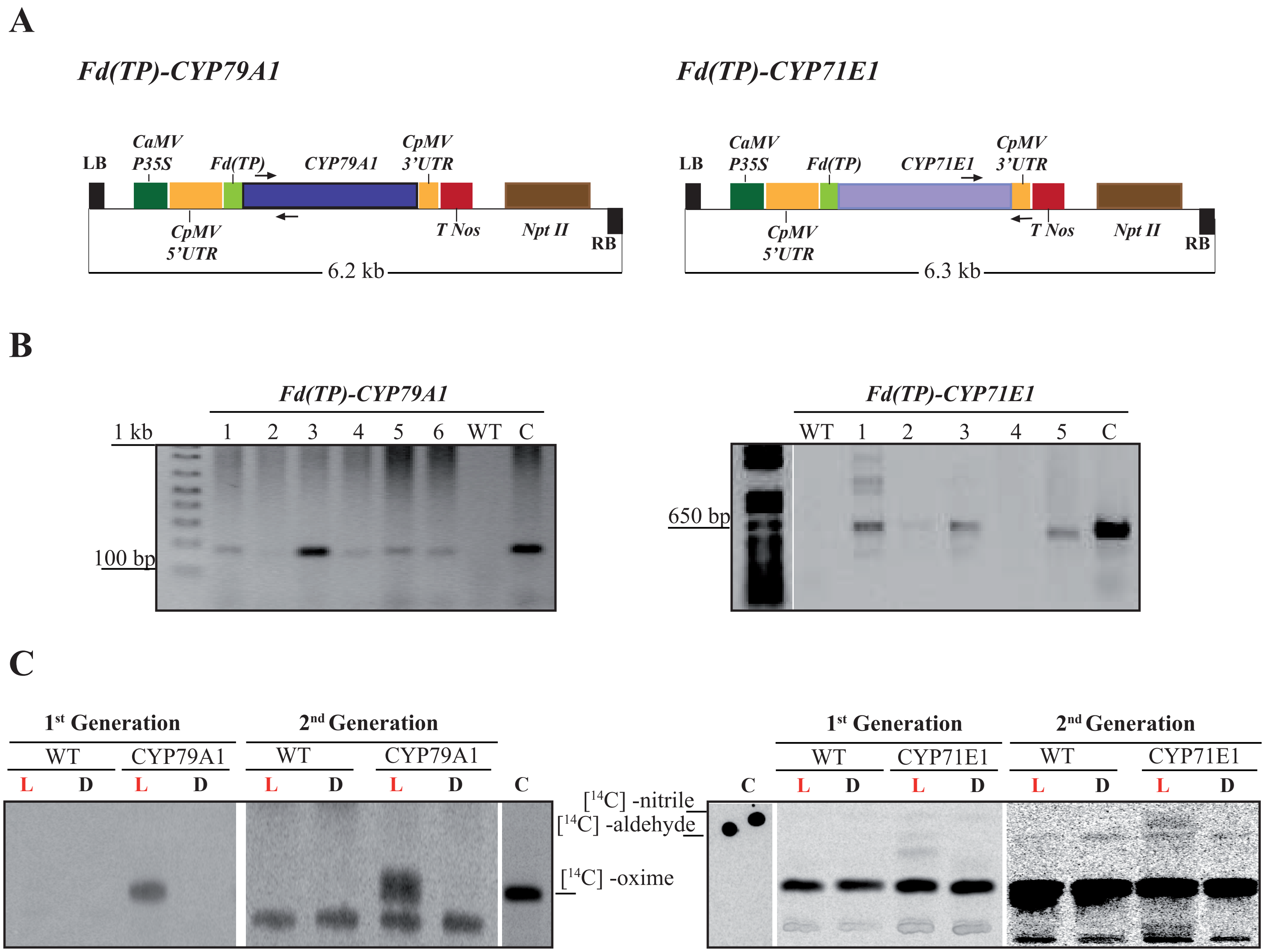

Fig.S2. Nuclear transformation of *N. benthamiana* with the genes encoding the two P450s involved in the dhurrin pathway. (A) Gene constructs encoding the two P450s fused to the ferredoxin transit peptide at the N-terminus (*Fd(TP)-CYP79A1* and *Fd(TP)-CYP71E1*) used for the nuclear integration in *N. benthamiana*. The genes are driven by the strong constitutive CaMV 35S promoter. (B) PCR analysis performed on genomic DNA extracted from the transformed lines: the *CYP79A1* engineered line (left) and the *CYP71E1* engineered line (right). The numbers 1-6 represent analysis of 6 independent transformed lines. “WT” indicates PCRs performed on genomic DNA extracted from the wild-type *N. benthamiana* and “C" indicates PCRs performed on plasmids harboring *Fd(TP)-CYP79A1* or *Fd(TP)-CYP71E1*. The primer binding sites are marked in small black arrows. (C) In vitro light-driven assay carried out with thylakoids isolated from the lines transformed with *CYP79A1* (left) and *CYP71E1* (right) genes. To assay CYP79A1 [^{14}C]-tyrosine was used as substrate and to assay CYP71E1 [^{14}C]-*p*-hydroxyphenylacetaldoxime (oxime) was used as substrate. The assay was carried out in the light (L) or the dark (D), and analyzed by TLC. After incubation, the assay mixtures were extracted with EtOAc and the organic phase was applied to the TLC.

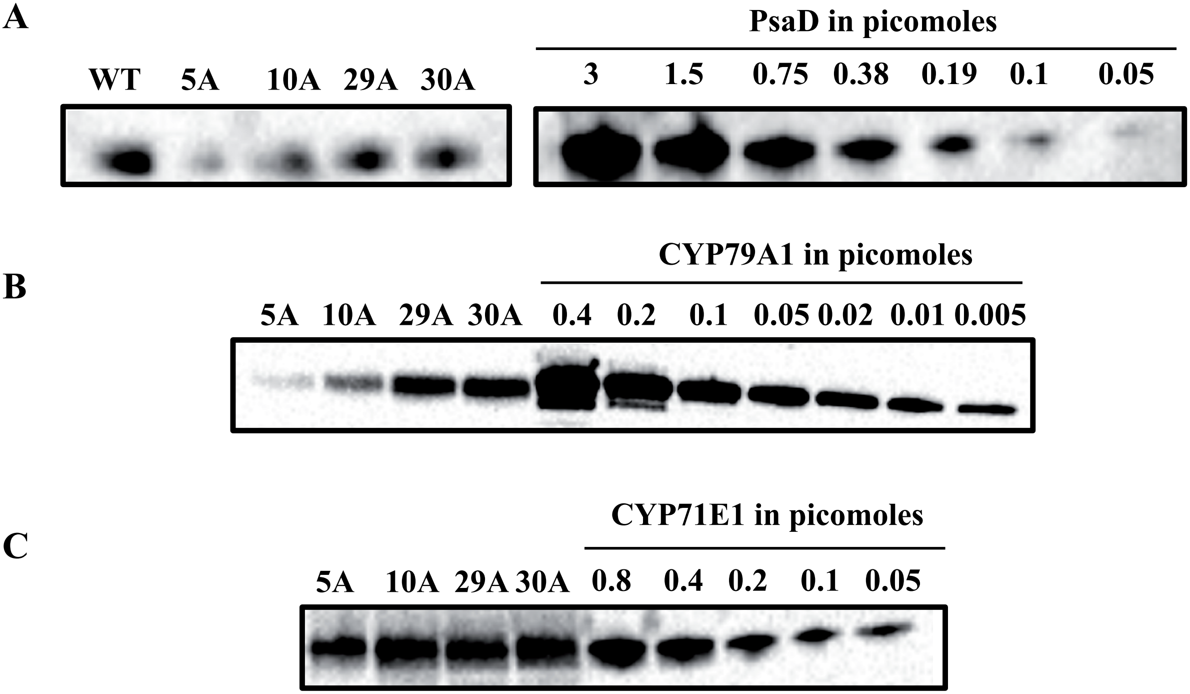

Fig.S3. The quantification of A) PsaD subunit B) CYP79A1 and C) CYP71E1 in the thylakoids of wildtype and the *DhuOp* lines. For quantification of PsaD subunit of PSI, thylakoid amount corresponding to 0.1 μg of total chlorophyll for the wildtype, 10A, 29A, 30 A lines and 0.03 μg of total chlorophyll for the line 5A was separated by SDS-PAGE, blotted and probed with PsaD primary antibody. For quantification of CYP79A1 and CYP71E1, thylakoids amount corresponding to 5μg and 2μg of total chlorophyll from the *DhuOp* lines, respectively. The proteins were separated by SDS-PAGE, blotted and probed with CYP79A1 and CYP71E1 primary antibodies. As standards, known concentrations of the purified PsaD fused to maltose binding protein, CYP79A1 and CYP71E1 were used. As the secondary antibody, polyclonal swine anti rabbit antibody was used.

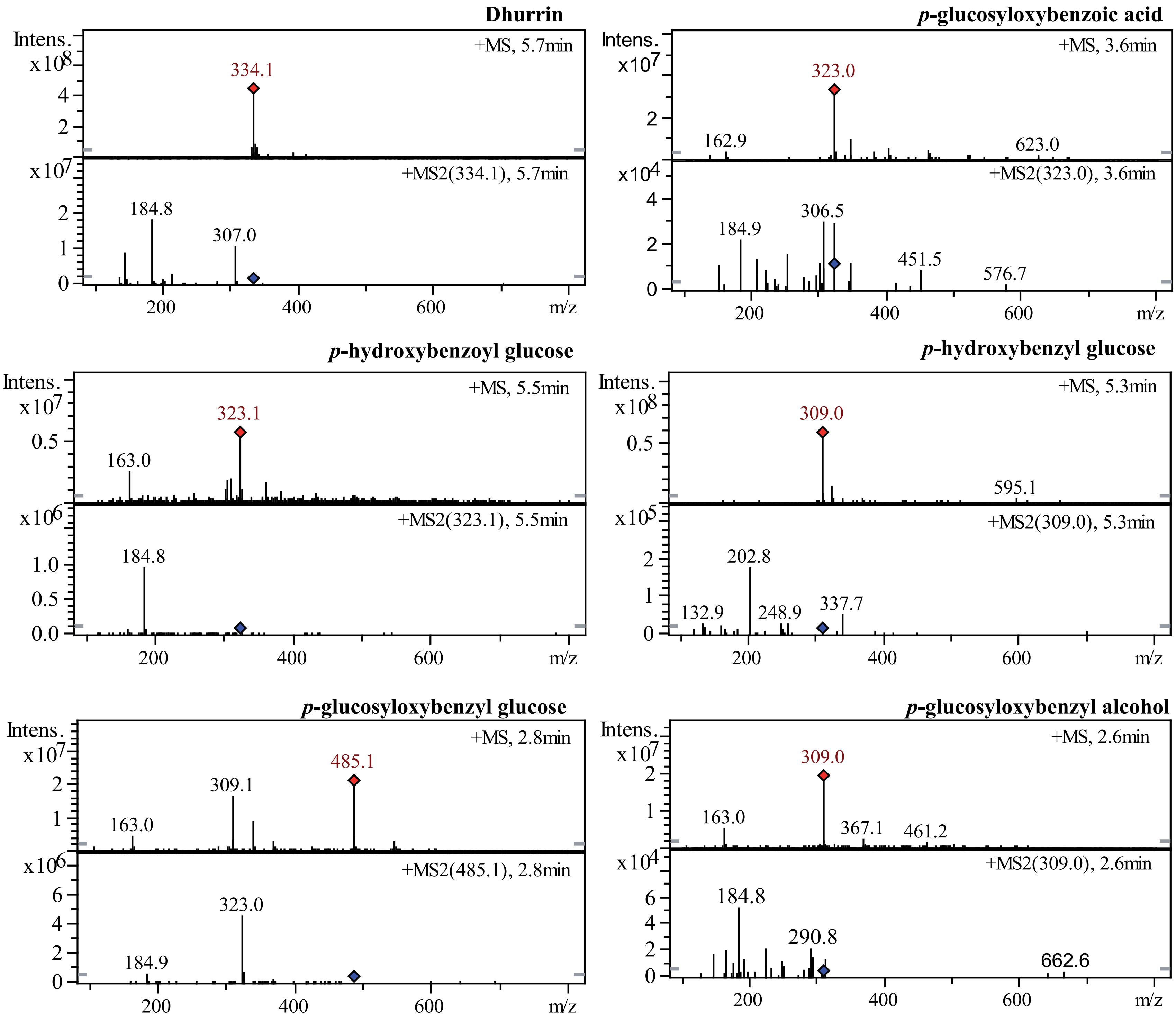

Fig.S4. Mass fragmentation pattern for dhurrin and selected glucosides produced from *p*-hydroxybenzaldehyde detected using LC-MS method.

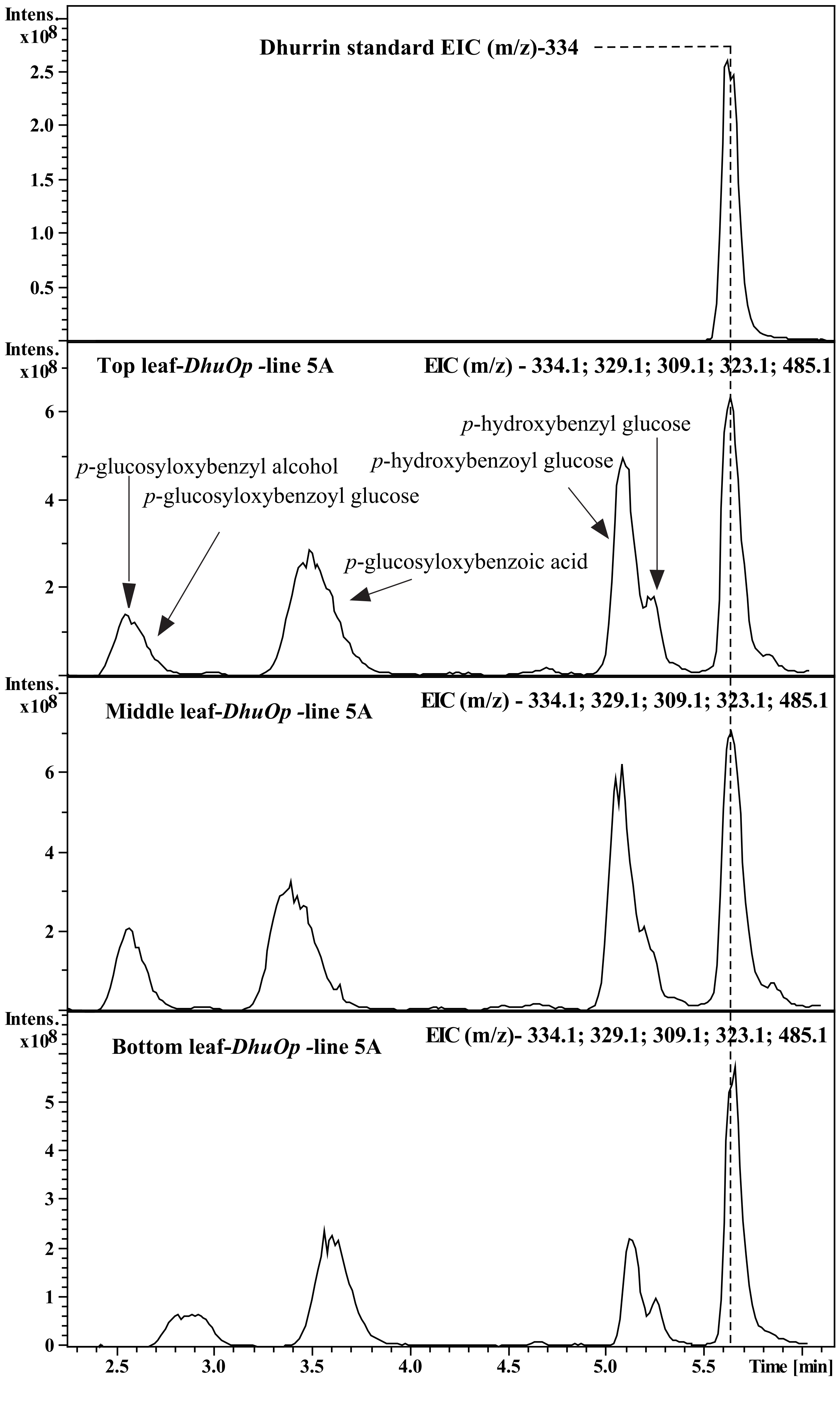

Fig.S5. LC-MS analysis of dhurrin and glucosides on the extracts from leaves of different age from the *DhuOp* line 5A. The top leaf represents the newly sprouted leaf (third leaf from the top), middle leaf represent the matured leaf, and the bottom leaf represents senescing leaves.
